# A Note on a Case of Functional Hemiplegia

**Published:** 1910-01-22

**Authors:** 


### Surgery.

### A NOTE ON A CASE OF FUNCTIONAL HEMIPLEGIA.

The case about to be described is one of more than ordinary interest, because it demonstrates the great deformity which may result from a hysterical cause even in quite young children, as well as the close similarity presented to a definite organic lesion.

History.

The patient, a girl aged eleven years, was quite healthy until six years ago, when she had what was, as far as can be ascertained, an attack of diphtheria. She recovered from the acute attack, but it was succeeded by paralysis of the palate and a slight weakness of the left triceps and the flexors of the left wrist. Finally the palatal paralysis recovered, but the weakness in the left arm, so far from improving, became more and more noticeable. In fact, she soon lost all use in it and carried it tightly flexed at the elbow with hyperextension of the wrist, and the fingers became useless, being kept flexed into the palm.

Later still she developed some general weakness of the left leg, but no single group of muscles appeared to be more affected than the others, so that no definite deformity resulted. She continued in this state until about a year ago, when she fell and hit her toe. After this she found she could not walk, and from this time onwards she was able to lie only on a couch; in fact, she was to all intents and purposes a helpless cripple.

Symptoms and Signs.

When the patient was first seen the most obvious point about her was that she was obviously of a nervous temperament, alternately crying and laughing during the course of the examination. This in itself was sufficient to give rise to a suspicion that the condition found might be largely, if not wholly, functional.

The left arm was held as described, fully flexed at the elbow and hyperextended at the wrist, the fingers being contracted into the palm.

The left leg was inverted, adducted strongly, and six centimetres shorter than the right one. There was some degree of talipes of an unusual variety—namely, equino-valgus. The foot could not be got up to a right angle. But on coming to examine the left hip there was at any rate an [illegible] organic lesion. The great trochanter was raised well above Nelaton's line, and the short side of Bryant's triangle was shorter on measurement than on the sound side. These facts, together with the shortening, inversion, and adduction of the limb, pointed to a dorsal dislocation. And that was what was actually found. The child was thin and the muscles somewhat flabby, so that the displaced head of the bone could be felt rotating with the limb in a position above and posterior to the acetabulum.

No anæsthesia nor hyperæsthesia could be discovered, and the nervous reflexes were normal, the knee-jerks being equal in both legs. There was no ankle clonus. Although there was definite atrophy, probably from disuse of the muscles of the left arm and leg, all the muscles reacting both to constant current and to faradic stimulation. The triceps and the flexors of the wrist reacted less strongly, but that was all.

At first sight the case appeared to be one of old hemiplegia with late spastic changes; but the hyperextension of the wrist did not correspond with this diagnosis, since in an organic lesion the flexors almost always overcome the extensors, and the hypothesis was put forward that it was functional. The truth of this was strikingly demonstrated when the child was told to perform movements for herself, *e.g.* extend the arm. At first she was quite unable to do so, but when told that if she couldn't the battery would have to be applied, she began to succeed immediately. Each joint was taken like this daily, with progressive improvement, and she now has a prospect of ultimately recovering complete use under continued treatment.

It must be supposed that after the diphtheria she had neuritis affecting certain groups of muscles slightly, and that the deformity which eventually resulted was the result of hysteria and nothing else. It is also possible that there was general weakness of the leg, which was allowed to progress. There can, of course, be little doubt that on the occasion when she fell she dislocated the left hip, an accident which was rendered less than ordinarily difficult of accomplishment by the lax and atrophic condition of the surrounding muscles.

The treatment of the arm was a much simpler affair than that of the leg, for in its case mere suggestion that she was quite able to use it, coupled with intimidation with a battery, was sufficient to get rid of the spasm and the loss of function. But in the case of the leg the dislocation had to be dealt with before any general improvement could be hoped for. The patient was accordingly anæsthetised, and an attempt was made to reduce the dislocation by manipulation. It was quite easy to get the head of the bone into the acetabulum with the leg in the flexed position, but as soon as the thigh was extended it immediately came out again. The head had apparently made its way through a rent in the capsule, which was very loose, and no amount of manipulation would persuade it to return by the way by which it came. Advantage was taken of the anæsthetic to test the real mobility of the ankle. It was found that the deformity was easily reduced without undue force. In fact, a noticeable feature of the case was the absolute absence of secondary changes in any of the joints, in spite of the fact that the spastic condition had lasted for so long a period. Manipulation having failed, there was no alternative but to reduce the hip by open operation, and for this a posterior incision was chosen. No difficulty was experienced, and the limb was put up on a splint. Under treatment on the lines already mentioned the spastic condition gradually cleared up, and there is now every hope that she may ultimately become almost as sound as she ever was.

